# Nicotine: Carcinogenicity and Effects on Response to Cancer Treatment – A Review

**DOI:** 10.3389/fonc.2015.00196

**Published:** 2015-08-31

**Authors:** Tore Sanner, Tom K. Grimsrud

**Affiliations:** ^1^Institute for Cancer Research, Norwegian Radium Hospital, Oslo University Hospital, Oslo, Norway; ^2^Department of Research, Cancer Registry of Norway, Oslo, Norway

**Keywords:** nicotine, tobacco, cancer, carcinogen, e-cigarettes

## Abstract

Tobacco use is considered the single most important man-made cause of cancer that can be avoided. The evidence that nicotine is involved in cancer development is reviewed and discussed in this paper. Both tobacco smoke and tobacco products for oral use contain a number of carcinogenic substances, such as polycyclic hydrocarbons and tobacco-specific *N*-nitrosamines (TSNA), which undoubtedly contribute to tobacco related cancer. Recent studies have shown that nicotine can affect several important steps in the development of cancer, and suggest that it may cause aggravation and recurrence of the disease. TSNA may be formed from nicotine in the body. The role of nicotine as the major addictive component of tobacco products may have distracted our attention from toxicological effects on cell growth, angiogenesis, and tumor malignancy. Effects on cancer disease are important aspects in the evaluation of possible long-term effects from sources of nicotine, such as e-cigarettes and products for nicotine replacement therapy, which both have a potential for life-long use.

## Introduction

Nicotine is the major chemical component responsible for addiction in tobacco products. At a high pH (alkaline), nicotine is in the non-ionized state and can be readily absorbed across the epithelium of the lung, the oral mucosa, the nose, and through the skin. A person smoking 25 cigarettes/day will absorb about 0.43 mg nicotine/kg bodyweight (1) and obtain a nicotine blood concentration in the range 4–72 ng/ml (0.025–0.444 μM) (2). The halftime of nicotine in plasma is about 2 h (3).

More than 80% of the nicotine absorbed undergoes metabolism in the liver, primarily by CYP2A6, UDP-glucuronosyltransferase, and a flavin-containing monooxygenase. Cotinine is the major metabolite. Nornicotine, formed by demethylation of nicotine is both a metabolite of nicotine and a minor tobacco alkaloid. As much as 85–90% of nicotine is metabolized before elimination via renal excretion (1). Cotinine blood concentration in smokers is 200–400 ng/ml (1.1–2.2 μM) (3).

Nicotine exerts its effects via stimulation of the nicotinic acetylcholine receptors (nAChRs), which are located in the CNS, at interganglionic junctions of the autonomic nervous system, and on target organs throughout the body as part of the parasympathetic autonomic nervous system. nAChRs are ligand-gated ion channels composed of five membrane-spanning subunits that combine to form a functional receptor (4). The homomeric α7-nAChR has been implicated as the primary receptor facilitating nicotine-mediated cell proliferation, but other receptor subunits may also be involved (5). Nicotine binds to nAChRs with a higher affinity than acetylcholine (Ach). It should be noted that tobacco-specific *N*-nitrosamines (TSNA) and cotinine may also bind to nAChRs (5–7).

The binding of nicotine to nAChR in the brain is involved in the rewarding effects of nicotine and in the adaptations that occur in response to chronic exposure, which give rise to dependence and to withdrawal responses. The positive reinforcing aspects of nicotine addiction primarily results from the release of dopamine (1).

Several lines of evidence indicate that nicotine may contribute to the development of cancer. Evidence from experimental *in vitro* studies on cell cultures, *in vivo* studies on rodents as well as studies on humans inclusive of epidemiological studies indicate that nicotine itself, independent of other tobacco constituents, may stimulate a number of effects of importance in cancer development (5, 6).

## Endogenous Formation of TSNA

**Figure 1:**
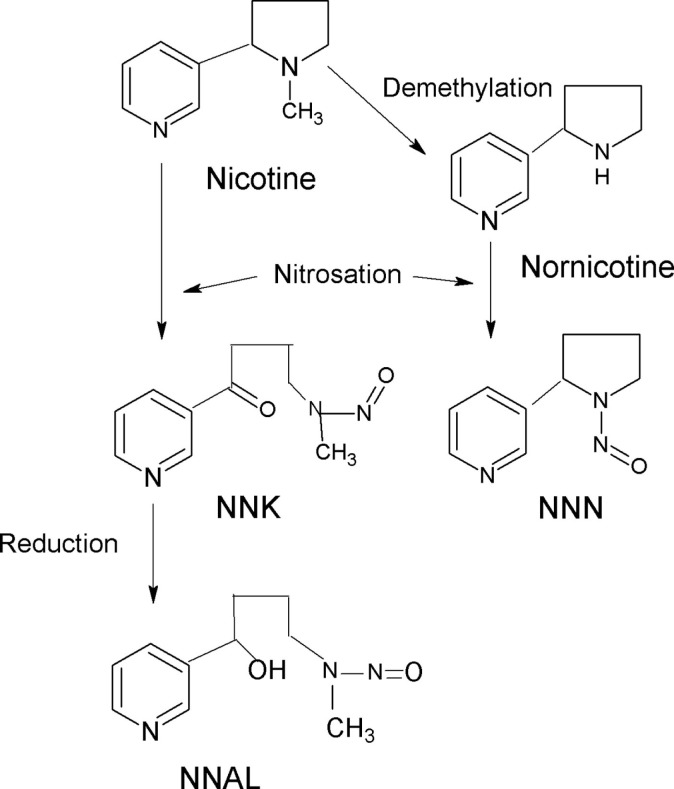
**Formation of NNK, NNN, and NNAL**.

Tobacco-specific *N*-nitrosamines is formed by *N*-nitrosation of alkaloids in tobacco (Figure 1). NNN (*N*′-nitrosonornicotine) and NNK (4-(metylnitrosamino)-1-(3-pyridyl)-1-butanon) are among the most important and most potent carcinogens in tobacco and tobacco smoke. NNAL (4-(methylnitrosamino)-1-(3-pyridyl)-1-butanol) is a metabolite of NNK and determination of total NNAL (NNAL and its glucuronides) in urine is often used to assess the possible role of TSNA in tumor development. A significant relationship between total NNAL has been found in serum samples from smokers and lung cancer risk (8). NNN and NNK are classified by International Agency for Cancer Research (IARC) as human carcinogens (9, 10).

It is well known that NNN and NNK are present both in mainstream and sidestream cigarette smoke and in moist snuff (11). In the 1996 review, Bartsch and Spiegelhalder (12) pointed out that *N*-nitrosamines may be formed endogenously in humans. Endogenous formation of TSNA has been demonstrated in rats treated with tobacco alkaloids and NaNO_2_ (13, 14).

It was claimed in some early studies that NNK is not formed *in vivo* (13, 15), but more recent data do not support this view. Thus, although the level of NNK in the Swedish snus is much lower than in the old type of snuff and in cigarette smoke (16), the level of total NNAL (a NNK metabolite) in urine from users of Swedish snus is still considerable, and only reduced to about half of that found in smokers and in users of old type snuff (Table 1). The level of total NNAL is also increased among users of nicotine replacement therapy (NRT) (17) compared with non-smokers exposed to ETS (18). It is of interest that the risk of cancer of the pancreas appears not to diverge materially between the different tobacco products (9). It has also been reported that NNN can be formed from nornicotine in human saliva (*in vitro*) (19). Recently, Hecht et al. (20) reported that the urine from users of e-cigarettes had very low levels of NNAL, which may suggest that endogenous formation of TSNA after nicotine inhalation is negligible. The above results indicate that TSNA may be formed endogenous after absorption of nicotine through the mucous membranes in the oral cavity and through the skin, while formation after lung absorption may be negligible. Thus, the toxicokinetics of nicotine may depend on the route of administration.

**Table 1:** **Level of NNAL in urine from smokers, users of “old” snuff in USA (smokeless tobacco), users of “modern” Swedish snus and of NRT compared to people exposed to ETS (17, 18, 21)**.

Group	Total NNAL (pmol/mg creatinine)
Smokers	2.6 (0.3–3.9)^a^
Snuff (old type)	3.3 (1.5–5.1)
Swedish snus	1.4 (0.9–2.0)
NRT	0.3 (0.1–0.4)
Non-smokers exposed to ETS	0.042 ± 0.020^b^

*^a^95% Confidence interval*.

*^b^±SD*.

## Genotoxicity of Nicotine

Most studies with the *Salmonella* assay (including urine of rats exposed to nicotine) were negative. However, nicotine has been shown to induce DNA damage in the *Escherichia colipol A*+*/pol*− test (22).

Two studies were positive for chromosomal aberration (CA) and sister chromatid exchange (SCE) in experiments with the CHO cell line (23, 24). These results have later been confirmed by Ginzkey et al. (25) in experiments with human lymphocytes. They found a significant increase in CA and SCE at a concentration of 1 μM nicotine (lowest concentration tested), which is only a factor of 2–3 higher than that found in the blood of smokers (2). DNA double-strand breaks (DSB) with missing repair are considered to be the most important lesion leading to observed CA.

In studies with the “Comet” assay, effects of nicotine has been reported in human nasal mucosa (26, 27), tonsillar tissue (28), cells of the parotid gland (29), and spermatozoa (30). On the other hand, Ginzkey et al. (25) did not observe any effect of nicotine in human lymphocytes after 24 h incubation. Since the Comet assay is known to detect DNA single-strand breaks (SSB), alkali-labile sites, and incomplete excision-repair sites in proliferating and non-proliferating cells, the authors argued that the lack of effects could be due to possible repair of DNA single-strand breaks during the 24 h incubation.

Argentin and Cicchett (31) studied formation of micronuclei (MN) by nicotine with human gingival fibroblasts and found that treatment with 1 μM nicotine significantly enhanced the frequency of MNs. Additions of antioxidants minimized the effect of nicotine by decreasing significantly the MN formation. Induction of MN was likewise found in a study with human lymphocytes (25), although a higher nicotine concentration was needed (100 μM). The mechanisms leading to the formation of MN are chromosome breakage and disturbance of the chromosome-segregation system, thus MN formation represents an irreversible DNA damage.

Adduct formation between nicotine and DNA has been reported by Cheng et al. (32). However, this has not been verified in a newer study by Hecht (33).

The mechanisms responsible for the genotoxic effects caused by nicotine have not been established. It is, however, of importance that effects are observed at concentrations of nicotine not much higher than those found in blood of smokers. The findings that the effects of nicotine are reduced in the presence of antioxidants suggest that oxidative radicals are involved. Moreover, the decrease in DNA damage reported after co-incubation with a nAChR antagonist indicates a receptor-dependent pathway for induction of oxidative stress (27).

## *In Vitro* Studies on Cell Cultures

### Signaling pathways

Low concentrations of nicotine stimulate cell proliferation, while high concentrations are cytotoxic (34). The concentrations of nicotine that stimulate cell proliferation correspond to the concentrations found in the bloodstreams of smokers and of users of oral tobacco. In this connection, it is of importance that nAChRs are also expressed on non-neuronal epithelial and endothelial cells and the nicotine stimulation of cell proliferation is inhibited by nAChR antagonists (35). It has been proposed that nicotine promotes cell division by upregulating of cyclin D1 (36).

The binding of nicotine and other nicotine metabolites to the nAChRs, stimulate signaling pathways and reactions that increase cell proliferation and cell survival. The nicotine-mediated secretion of epidermal growth factor (EGF) via nAChRs results in transactivation of epidermal growth factor receptor (EGFR) and activation of mitogenic and antiapoptotic pathways (37, 38). The nicotine-mediated release of adrenaline and noradrenaline, which are the physiologic ligands for β-adrenergic receptors (β-ARs), leads to the binding to and activation of β-AR. This results in stimulation of multiple oncogenic and mitogenic signaling cascades, which activates proliferative pathways and the release of EGF, vascular endothelial growth factor (VEGF), and arachidonic acid (39–41). In addition, it has been found that nicotine itself binds to β-ARs (39).

Nicotine induces epithelial–mesenchymal transition (EMT), which is one of the vital steps for the acquisition of malignant phenotype. This transition allows the cell to acquire migratory properties, which may facilitate cancer metastases (42).

Nicotine decreases the tumor suppressor Chk2, which is activated by DNA damage. The decrease in Chk2 in cells exposed to nicotine suggests that nicotine may be capable of overriding DNA damage checkpoint activation, disrupting genetic surveillance, and increasing the risk of oncogenesis (43).

### Angiogenic growth

Nicotine promotes endothelial cell migration, proliferation, survival, tube formation, and nitric oxide (NO) production *in vitro*, mimicking the effect of other angiogenic growth factors (44, 45). In 2001, it was found that nicotine was a potent angiogenic agent at tissue and plasma concentrations similar to those induced by light to moderate smoking (46). Effects of nicotine on angiogenesis have been demonstrated for a number of tumor cells, such as breast, colon, and lung (47, 48). Similar results have also been demonstrated in *in vivo* mouse models of lung cancer, where nicotine significantly increased the size and number of tumors in the lung, and enhanced metastasis (49).

### Interference with cancer therapy

In several *in vitro* experiments, it has been found that nicotine in concentrations as low as 1 μM decreased the anti-proliferative and pro-apoptotic effects exerted by chemotherapeutics on several different malignant cell lines (50–52). These effects were partially reverted by exposure to α-bungarotoxin (α-BTX), an inhibitor of α7-nAChR (51). In the case of radiotherapy (RT), nicotine administration increased survival of H460 and A549 lung cancer cells. This effect was likewise reduced by addition of α-BTX prior to nicotine addition and radiation (53). On this basis, it is expected that use of nicotine products during cancer treatment may reduce the effects due to reactions following interaction of nicotine with α7-nAChR.

It was found in 1998 (54) that nicotine activates the mitogen-activated protein (MAP) kinase signaling pathway in lung cancer cells. This results in increased expression of the *bcl*-2 protein and inhibition of apoptosis. These effects may also contribute to reduction of the effect of chemotherapy in smokers (44).

### Cotinine

Cotinine was found to increase significantly proliferation of the human lung adenocarcinoma A549 cells at a concentration of 0.1 μM. The effect was abolished by the phosphoinositide 3-kinase inhibitor LY294002 (55). Moreover, it was found that cotinine in a concentration of 0.01 μM inhibited doxorubicin-induced apoptosis by suppressing caspase-mediated apoptosis. Caspases or cysteine-aspartic proteases are essential for programed cell death. The results suggested that cotinine suppressed apoptosis through the PI3K/Akt pathway just as nicotine does.

## *In Vivo* Studies on Rodents

### Carcinogenicity studies with nicotine

A group of 68 female Sprague-Dawley rats was exposed 20 h 5 days/week to nicotine by inhalation (56). The control group consisted of 34 animals. The nicotine concentration in air was 500 μg/m^3^ (nicotine concentration in plasma slightly above 100 ng/ml in exposed rats). Forty-four (65%) exposed rats and 17 (50%) controls were alive after 1 year and 30 (44%) exposed and 9 (26%) controls after 1.5 years. At the end of the study (after 24 months) the remaining rats, 22 (32%) nicotine exposed animals and 7 (21%) controls – were sacrificed and examined for tumors. The fraction of tumors in the pituitary gland was higher in the exposed group (5/59 versus 0/25). However, it is difficult to evaluate the results as the time of tumor identification is not given. Moreover, due to the low sensitivity of animal experiments, the doses used in animal experiments to evaluate potential carcinogenic effects are usually several times higher than those that human may be exposed to. Normally the highest dose used in an animal experiment should result in a toxicity giving about 10% decreased weight. In the present experiment, the plasma nicotine level was only slightly higher than that of smokers, and the decrease in weight at 24 months was only 3% in the exposed animals compared to the controls. Thus, the exposure doses used and the missing of important information in the paper prevent the drawing of firm conclusions from the experiment.

Female A/J mice received subcutaneous (s.c.) injections of nicotine hydrogen tartrate (3 mg/kg bw/day, 5 days/week for 24 months), while a control group received saline injections (57). The study showed that 73% of the nicotine-treated mice but none of the control mice developed neoplasms originating from the uterus or skeletal muscle. The uterine tumors were diagnosed as leiomyosarcoma, and those in quadriceps as rhabdomyosarcoma. No metastases were observed. Rhabdomyosarcoma can develop spontaneously in A/J mice, but leiomyosarcoma does not (58), which indicates that the leiomyosarcoma development was specific to the experimental nicotine treatment. Thus, the experiment may suggest that nicotine is a complete carcinogen.

Male Syrian golden hamsters maintained in 60% hyperoxia and receiving s.c. injections of nicotine for the duration of their life (up to 64 weeks) developed a low but significant number of tumors (2/16 adenocarcinoma of the nasal cavity, one of these hamsters had also an adenocarcinoma of the adrenal gland, and 2/16 carcinomas and 2/16 adenomas of the lung) (59). No metastases were observed. Hamsters receiving nicotine and maintained in ambient air or given saline injections and maintained in 60% hyperoxia did not develop tumors in any organs.

Addition of nicotine hydrogen tartrate in the drinking water of female Wistar Han rats and female C57Bl/6 mice for 4 weeks induced hyperplasia of the urinary bladder epithelium (60). These results may be consistent with induction of an early-stage carcinogenicity; however, no conclusion can be drawn from this 4-week study.

Except for the inhalation experiment by Waldum et al. (56), no long-term animal experiments of the type normally used to evaluate carcinogenicity are available for nicotine. Due to several shortcomings, no conclusion can be drawn from this study. Results from experiments with injections are generally considered to be of less importance in evaluation of carcinogenicity unless the tumors induce metastases. Thus, at present it is not possible to draw any conclusion with regard to the potential carcinogenic effect of long-term treatment with nicotine.

### Cocarcinogenicity and promoter activity

Nicotine showed cocarcinogenic effect with DMBA (7,12-dimethylbenz[a]anthracene) in the hamster cheek pouches model (61) and promoter activity with MNNG (*N*-methyl-*N*′-nitro-*N*-nitrosoguanidine) as initiator in a rat stomach model (62). In the above experiments, no effect was found when treated with nicotine alone.

No effects of nicotine were found with NMU (*N*-nitrosomethylurea) as an initiator in a rat mammary tumor model. However, 100% of the animals treated with NMU alone developed tumors (63). It was reported in a rat experiment (64) with hormone-dependent autochthonous mammary carcinomas induced by HECNU, a water-soluble nitrosourea, that nicotine had an antitumor effect. Both *trans*-*nicotine-N*′*-oxide* and a mixture of *cis*- and *trans*-nicotine-*N*′-oxides promoted induction of forestomach tumors in the rat, but not urinary bladder tumors after initiation with FANFT (*N*-[4-(5-nitro-2-furyl)-2-thiazolyl]formamide). Cotinine was also tested as promoter, but had no effect (65).

### Initiation by NNK

Five experiments have been identified in mice with NNK (100 mg/kg bw, weekly in 1–5 weeks) as an initiator and nicotine as a promoter. Nicotine was administered by i.p. injections (1 mg/kg bw, three times a week for 10–28 weeks) in two experiments (49, 66) and in the drinking water (0.07–0.1 mg/ml) for 12–44 weeks in three experiments (55, 67, 68). Nicotine showed promoter activity in the two experiments after i.p. injection, while only a small non-significant or no promoter activity was found after nicotine in the drinking water. It is unlikely that the difference in the effect of nicotine is due to the administration of the initiator NNK, as the dose per injection was the same in all experiments. Moreover in the experiments with i.p. injection of nicotine, the number of NNK injections was only one in the experiment of Iskandar et al. (66). In the drinking water experiments, the mice received 1–3 NNK injections. Moreover, it is unlikely that the difference can be accounted for by the time span of the promotion period, which ranged from 10 to 28 weeks for the i.p. injection experiments and from 12 to 44 weeks in the drinking water experiments. Thus, the different results are most likely due to the route of administration of nicotine, as the parietal part of the peritoneum drains directly to the systemic circulation.

The levels of nicotine and cotinine in blood and urine have only been measured in a few studies. The most interesting observation is the low level of nicotine and cotinine in serum observed by Murphy et al. (68) after addition of nicotine to the drinking water. They found 0.26 ng/ml of nicotine and 29 ng/ml of cotinine. The ratio of cotinine/nicotine is thus about 100. No serum values are available from the other studies. However, Zhou et al. (69) measured nicotine and cotinine in serum after i.p. injection of 1 mg/kg bw in CYP2A5 WT mice. They found a nicotine level of 45 ng/ml, which decreased to 0 after 60 min and a cotinine level 300 ng/ml, which decreased to 0 after 240 min. The ratio of cotinine/nicotine is thus about 5 and the level of nicotine was more than 100 times higher than that observed by Murphy and coworkers (68). The cotinine concentration after human use of tobacco products are in general 10 times or less higher than the nicotine concentration when measured simultaneously in serum or other tissues (1, 70). The reason for the large difference may be that the drinking water experiments have a considerable first-pass metabolism of nicotine in liver before it enters the systemic circulation and less control of how much the mice were drinking and thus their total nicotine intake. Matta and coworkers (71) point out that only about 30% of orally administered nicotine can be expected to reach the circulation, while the other 70% are metabolized primarily to cotinine before reaching the blood stream. The results of the measurements immediately after i.p. injections of nicotine were similar to that found among smokers of about 25 cigarettes/day. The lacking effects of nicotine in the drinking water experiments may thus be related to low serum nicotine levels.

Most of the experiments involving promotion after initiation with NNK have been carried out with A/J mice as this strain is highly susceptible to lung carcinogens, while C3H and C57BL/6 mice are relatively resistant. The mechanism(s) of different susceptibility are not known and may be multifactorial. It has been reported that NNK causes a significant increase in the expression of α7-nAChRs and COX-2 in the A/J lung, which might contribute to its higher susceptibility to NNK-induced lung tumorigenesis (72).

### Effects on tumor progression

Six experiments related to the effect of nicotine on tumor progression have been identified. In these experiments, malignant cells were injected in mice and the mice were subsequently treated with nicotine. In two experiments where nicotine was administered in drinking water, no effect of nicotine was observed. In the first study by Jarzynka et al. (73), ovariectomized nude mice received nicotine (0.2 mg/ml drinking water) for 5 weeks after implantation of human A549 bronchioloalveolar carcinoma cells. If the mice also received estradiol, the tumor sizes increased significantly. In the other experiment by Maier et al. (67), AB6F1 mice received nicotine 0.1 mg/ml in drinking water or i.p. injection with nicotine 0.8 mg/kg bw three times a week for 2–5 weeks after injection of CL13, IO33, or CL25 cells (all cell lines were derived from a lung adenocarcinoma). Neither i.p. injection of nicotine nor nicotine drinking water did enhance tumor growth or formation of metastases. The authors suggested that nicotine has a dose threshold, below which it has no appreciable effect.

On the other hand, Davis et al. (49) implanted subcutaneously Line1 mouse adenocarcinoma cells into syngenic BALB/c mice and observed that subsequent administration of nicotine by i.p. injections or transdermal patches enhanced tumor growth, metastases formation and tumor recurrence. The authors pointed out that mice receiving nicotine by i.p. injections had an average cotinine concentration of 3 μg/ml in urine and those that received nicotine by transdermal patch had an average cotinine concentration of 5 μg/ml. In human smokers, cotinine concentrations in urine have been reported in values ranging from 1.5 to 8.0 μg/ml. Al-Wadei et al. (74) inoculated male athymic nude mice by s.c. injection with Panc-1 cells (cell line isolated from a human pancreas cancer) and found that 0.2 mg/ml nicotine in drinking water increased the xenograft volumes significantly. In a study with Lewis *in vivo* lung cancer model, Nakada et al. (55) reported that both nicotine (0.1 mg/ml) and cotinine (0.1 mg/ml) in drinking water significantly increased tumor growth after implementation of Lewis lung carcinoma cells in mice. Moreover, both nicotine and cotinine accelerated capillary formation of vascular endothelial cells. Heeschen et al. (46) studied if nicotine could enhance tumor angiogenesis in the Lewis lung cancer model. Sixteen days after implantation of the cancer cells and treatment with nicotine (0.1 mg/ml), tumor growth in the nicotine group markedly exceeded that in the vehicle-treated group and required killing of the mice. This acceleration of tumor growth in the nicotine group corresponded with increased vascularization of the tumor tissue. In a subsequent experiment, it was found that antagonists of the nAChR abolished the proangiogenic effect of nicotine. Nicotine enhanced tumor progression in four of the six experiments. Enhancements were found both after exposure of nicotine by i.p. injection, oral, and skin administrations. Moreover, cotinine did also enhance tumor growth.

### Reduced protection from cancer immunosurveillance

Smokers are less responsive to vaccines (75, 76). However, the mechanism for this effect on host immunity is not known. A general immunosuppressive effect of nicotine was shown by decreased interleukin-2 (IL-2) production in mitogen-stimulated human peripheral blood mono-nuclear cells (77). Nouri-Shirazi and Guinet (78) demonstrated that animals exposed to nicotine produced an inadequate effector/memory T cell population in response to protein-based vaccine formulated with Th1 adjuvants. In addition, the prime-boost vaccination recalled insufficient memory response and failed to protect the animals from an otherwise prophylactic and therapeutic vaccine. It has also been reported that exposure to nicotine adversely affects dendritic cells, a cell type that has an important role in anticancer immunosurveillance (79).

### Chemotherapy and radiotherapy

As early as 1988, Berger and Zeller (80) reported that the antitumor effect of cyclophosphamide (CPA) on transplanted rat L5222-leukemia was reduced by administration of nicotine. However, using a mammary carcinoma model, it was found that administration of nicotine resulted in greater tumor inhibition than the antitumor drug HECNU alone. The authors pointed out that further combination studies with other classes of cytotoxic drugs are warranted.

Warren et al. (53) studied the role of nicotine on response to RT and chemoradiotherapy (CRT) *in vivo*. Male athymic nude Foxn1^nu^ mice were inoculated with H460 human lung cancer cells to form single xenografts in the right rear flank. Nicotine administration during fractionated RT or CRT (5-day treatment) increased xenograft regrowth as compared to RT or CRT alone. The observation that short-term nicotine (every other day for 6 days) produced similar tumor regrowth curves as long-term nicotine (every other day during treatment, maximum 28 days) further suggests that nicotine exposure specifically during treatment is the critical determinant of therapeutic outcome. Further analysis demonstrates that nicotine appears to increase HIF-1α (hypoxia-inducible factor 1, alpha subunit) expression *in vivo* with no change in a clinical marker of tumor hypoxia (immunohistochemical CAIX expression). The authors emphasized that the *in vivo* effects of nicotine on therapeutic response support nicotine as an important systemically available component of tobacco for decreasing the efficacy of cancer treatments.

Banerjee et al. (52) inoculated subcutaneously male athymic nude mice in the flank region with pancreatic ductal adenocarcinoma BXPC-3 cells. The mice were treated either with 50 mg/kg bw gemcitabine twice a week by i.p injections, 1 μM nicotine in the drinking water or with both nicotine in the drinking water and gemcitabine i.p. injections. All treatments started 1 day after s.c. inoculation of the tumor cells and the animals were observed for 30 days. Treatment of mice with gemcitabine alone reduced xenograft volumes in weeks 2–4 with 20%. Nicotine treatment significantly (*p* < 0.001) reduced this therapeutic effect of gemcitabine in weeks 2–4). In accord with the documented ability of gemcitabine to induce apoptosis, BXPC-3 xenografts of the animals treated with gemcitabine alone showed induced protein levels of cleaved caspase-3. By contrast, the induction of cleaved caspase in response to gemcitabine was completely abolished by nicotine (*p* < 0.001).

Due to the effects of nicotine during *in vivo* chemotherapy and RT, concern of the use of NRT in relation to cancer treatment has been expressed (52, 53). The advantage of NRT is that it does not contribute the large number of carcinogens present in tobacco smoke (53).

## Effects on Humans

To our knowledge, there are no relevant study in humans on carcinogenic effects from pure nicotine including products, such as NRT and e-cigarettes. Observational studies on smokers and users of oral tobacco may still provide important suggestions on potential effects – as exposures to some of the tobacco-specific constituents may be similar. Nicotine is a tobacco component where blood concentrations are similar during smoking and use of oral tobacco (81). Therefore, relevant effects from tobacco use in humans will be briefly reviewed.

### Use of tobacco prior to cancer diagnoses

A higher risk of death from cancer among current users of tobacco than among never users, which include both smoking and oral smokeless tobacco, has been demonstrated for a number of different types of cancer in the literature [see, e.g., Ref. (82–93)]. Only a few studies will be discussed in the present section.

Nordenvall et al. (90) studied the use of tobacco and risk of death in cancer patients among 336,381 Swedish construction workers. A total of 40,230 cases of cancer were identified. Hazard ratios and 95% confidence intervals (CIs) for death from any cause, cancer-specific death and death from other causes were analyzed. Never users of any tobacco served as reference. Increased risks of cancer-specific death were observed both among exclusive smokers (HR = 1.15, 95% CI: 1.10–1.21) and never-smoking snus users (HR = 1.15, 95% CI: 1.05–1.26). It should be noted that in the case of snus users, the major part of nicotine is absorbed through the oral mucosa. The authors pointed out that nicotine may be the common cause to explain these findings.

It is of interest that the effects are not limited to patients suffering from tobacco-related cancers. Although smoking is not likely to be a risk factor for initiating prostate cancer, men who are smoking cigarettes before diagnosis appear to have a worse prognosis (87, 89, 93, 94). Wilson et al. (93) recently reported in an abstract a study on 9582 men with prostate cancer among Swedish construction workers. Compared to never users of tobacco, exclusive smokers were at increased risk of both prostate cancer mortality (HR = 1.15, 95% CI: 1.05–1.27) and total mortality (HR = 1.17, 95% CI: 1.09–1.26). Exclusive snus users also had increased risks for both prostate cancer mortality (HR = 1.24, 95% CI: 1.03–1.49) and total mortality (HR = 1.19, 95% CI: 1.04–1.37). The authors concluded that the results suggest that nicotine may promote cancer progression independent of the combustion products of tobacco smoke.

### Use of tobacco after diagnosis and during cancer treatment

People who have used tobacco after a cancer diagnosis and during cancer treatment have also used tobacco prior to their diagnoses. In studies where survival or recurrence have been assessed, it is difficult to differentiate between the contribution of tobacco use prior to diagnoses and during cancer treatment. Moreover, the studies are primarily on people smoking and only to a limited extent on other types of nicotine-containing substances, such as oral tobacco. Some studies where it is specifically stated that the patients smoked during treatment are discussed below.

It is well known that smoking has an adverse effect in relation to surgery. This has also been demonstrated for cancer surgery. An example is the study of Sørensen et al. (95) on 425 patients that underwent different forms of breast cancer surgery as simple mastectomy, modified radical mastectomy, or breast conserving surgery in a hospital in Denmark. The authors concluded that independent of other risk factors, smoking is predictive for post-mastectomy wound infection, skin flap necrosis, and epidermolysis. The effects were in all cases larger for heavy smoking than for light smoking.

In a study of prostate cancer, patients who continued to smoke after radical prostatectomy had a higher recurrence than former smokers (34.3 versus 14.8%) (96). Steinberger et al. (97) followed 2358 patients receiving external beam radiotherapy (EBRT) between 1988 and 2005 for clinically localized prostate cancer. Current smoking significantly increased the risks of both prostate-specific antigen relapse [HR = 1.37 (1.04–1.84)], distant metastases [HR 2.30 (1.57–3.36)], as well as prostate cancer-specific death [HR = 2.25 (1.30–3.88)]. Moreover, current and former smokers, regardless of duration and quantity of exposure, had an increased risk of long-term genitourinary toxicity after EBRT.

It is agreed that smoking cessation during treatment for cancer does in general result in better response and increased survival (98). This conclusion is based both on *in vitro* and *in vivo* experiments as well as epidemiological studies. Nicotine may be involved in these effects.

## Conclusion

All tobacco products contain various amounts of carcinogenic substances, such as polycyclic hydrocarbons (PAH) and TSNA, which undoubtedly play an important role in development of cancer. Evidence from experimental *in vitro* studies on cell cultures, *in vivo* studies on rodents as well as studies on humans inclusive of epidemiological studies indicate that nicotine may contribute in cancer development by stimulating a number of important processes. Nicotine acts primarily by activation of nicotine acetylcholine receptors (nAChRs) and nicotine binds to these receptors with a higher affinity than acetylcholine. Furthermore, the TSNA substances NNN (*N*′-nitrosonornicotine) and NNK (4-(metylnitrosamino)-1-(3-pyridyl)-1-butanon) may be formed from nicotine after oral administration. The role of nicotine in carcinogenesis is of great importance in the evaluation of potentially harmful effects from non-tobacco related sources of nicotine, such as e-cigarettes and NRT.

Nicotine has been shown to induce CA, SCE, single-strand DNA strand breaks, and MN *in vitro*. Oxidative stress is probably involved since the effects are reduced in the presence of antioxidants. The finding that the effects decrease after co-incubation with a nAChR antagonist indicates a receptor-dependent pathway for induction of oxidative stress.

The interaction of nicotine with nAChRs activates signaling pathways that result in a number of reactions, such as increased cell proliferation and cell survival. Although nAChRs are the primary receptors, binding of nicotine to β-ARs and EGFRs may also be important. Nicotine induces EMT, which is involved in the acquisition of malignant phenotype. Moreover, nicotine induces changes that mimic the effects of angiogenic growth factors.

At present, it is not possible to draw a conclusion whether nicotine itself may act as a complete carcinogen. In mice studies with NNK as an initiator, nicotine acts as a promoter after injection or dermal absorption, but not after oral administration. In drinking water experiments, there is considerable first-pass metabolism of nicotine before nicotine enters the systemic circulation. As a result, serum concentration is much lower after ingestion than after i.p. administration. Nicotine enhanced tumor growth and progression after injection of malignant cells in mice. Enhancements were found both after exposure of nicotine by i.p. injection, oral, and skin administration. Moreover, cotinine did also enhance tumor growth.

Nicotine may inhibit antitumor immune response. It has also been reported that exposure to nicotine adversely affects dendritic cells, a cell type that has an important role in anticancer immunosurveillance. Moreover, in studies on xenograft in mice, nicotine has been found to reduce the effect of RT and CRT.

Reduced overall survival and specific disease survival after cancer diagnoses have been found among current smokers compared with never smokers, as well as in users of smokeless tobacco, such as snus, even for cancers thought to be unrelated to tobacco. Although more studies on health effects of nicotine in humans are required, based on *in vitro* and *in vivo* effects of nicotine, patients should be advised not to use nicotine products during cancer treatment unless it is temporarily needed to stop tobacco smoking.

## Conflict of Interest Statement

The authors declare that the research was conducted in the absence of any commercial or financial relationships that could be construed as a potential conflict of interest.

## Abbreviations

α-BTX, α-bungarotoxin; β-AR, β-adrenergic receptor; Ach, acetylcholine; CA, chromosomal aberration; CI, confidence interval; CPA, cyclophosphamide; CRT, chemoradiotherapy; DMBA, 7,12-dimethylbenz[a]anthracene; DSB, double-strand breaks; EBRT, external beam radiotherapy; EGF, epidermal growth factor; EGFR, epidermal growth factor receptor; EMT, epithelial–mesenchymal transition; FANFT, *N*-[4-(5-nitro-2-furyl)-2-thiazolyl]formamide; HR, hazard ratio; IARC, International Agency for Cancer Research; MAP, mitogen-activated protein; MNNG, *N*-methyl-*N*′-nitro-*N*-nitrosoguanidine; nAChRs, nicotinic acetylcholine receptors; MN, micronuclei; NMU, *N*-nitrosomethylurea; NNAL, 4-(methylnitrosamino)-1-(3-pyridyl)-1-butanol; NNK, 4-(metylnitrosamino)-1-(3-pyridyl)-1-butanon); NNN, *N*′-nitrosonornicotine; NRT, nicotine replacement therapy; PAH, polycyclic hydrocarbons; RT, radiotherapy; SCE, sister chromatide exchange; SSB, single-strand breaks; TSNA, tobacco-specific *N*-nitrosamines.
